# Recognition of parasitic helminth eggs via a deep learning-based platform

**DOI:** 10.3389/fmicb.2024.1485001

**Published:** 2024-11-20

**Authors:** Wei He, Huiyin Zhu, Junjie Geng, Xiao Hu, Yuting Li, Haimei Shi, Yaqian Wang, Daiqian Zhu, Huidi Wang, Li Xie, Hailin Yang, Jian Li

**Affiliations:** ^1^Key Laboratory of Industrial Biotechnology, Ministry of Education, School of Biotechnology, Jiangnan University, Wuxi, China; ^2^Department of Human Parasitology, School of Basic Medicine Science, Hubei University of Medicine, Shiyan, China; ^3^Department of Pediatrics, Taihe Hospital, Hubei University of Medicine, Shiyan, China; ^4^School of Internet of Things Engineering, Jiangnan University, Wuxi Jiangsu, China; ^5^BRC Biotechnology (Shanghai) Co. Ltd., Shanghai, China

**Keywords:** human parasites, egg, artificial intelligence, YOLO model, deep learning, rapid diagnosis

## Abstract

**Introduction:**

Accurate and rapid diagnosis is crucial for the effective treatment of parasitosis. Traditional etiological methods, especially microscopic examination, are time-consuming, labor-intensive, and prone to false or missed detections. In response to these challenges, this study explores the use of artificial intelligence (AI) for the detection and classification of human parasite eggs through the YOLOv4 deep learning object detection algorithm.

**Methods:**

Eggs from species such as *Ascaris lumbricoides* (*A*. *lumbricoides*), *Trichuris trichiura* (*T*. *trichiura*), *Enterobius vermicularis* (*E*. *vermicularis*), *Ancylostoma duodenale* (*A*. *duodenale*), *Schistosoma japonicum* (*S*. *japonicum*), *Paragonimus westermani* (*P*. *westermani*), *Fasciolopsis buski* (*F*. *buski*), *Clonorchis sinensis* (*C*. *sinensis*), and *Taenia* spp. (*T*. *spp*.) were collected and prepared as both single species and mixed egg smears. These samples were photographed under a light microscope and analyzed using the YOLO (You Only Look Once) v4 model.

**Results:**

The model demonstrated high recognition accuracy, achieving 100% for *Clonorchis sinensis* and *Schistosoma japonicum*, with slightly lower accuracies for other species such as *E*. *vermicularis* (89.31%), *F*. *buski* (88.00%), and T. trichiura (84.85%). For mixed helminth eggs, the recognition accuracy rates arrived at Group 1 (98.10, 95.61%), Group 2 (94.86, 93.28 and 91.43%), and Group 3 (93.34 and 75.00%), indicating the platform’s robustness but also highlighting areas for improvement in complex diagnostic scenarios.

**Discussion:**

The results show that this AI-assisted platform significantly reduces reliance on professional expertise while maintaining real-time efficiency and high accuracy, offering a powerful tool for the diagnosis and treatment of parasitosis. With further optimization, such as expanding training datasets and refining recognition algorithms, this AI system could become a key resource in both clinical and public health efforts to combat parasitic infections.

## 1 Introduction

Parasitic diseases rank among the most devastating and prevalent infectious diseases worldwide, affecting nearly two billion people with soil-transmitted helminths such as *Ascaris lumbricoides*, *Ancylostoma duodenale*, *and Trichuris trichiura*. In China, approximately 5.96% of the population suffers from these infections, with a total of around 38.59 million cases, predominantly due to helminths (Simarro et al., 2011; Momčilović et al., 2019). The effective management of parasitic diseases hinges on accurate and rapid diagnosis, which is essential for timely treatment and control measures.

Currently, the use of manual microscopy to find parasitic eggs, larva, cyst, oocyst, or trophozoite as a low-cost test is still the gold standard for parasite diagnosis, which is widely used in clinical practice, especially in economically underdeveloped areas (Ricciardi and Ndao, 2015). The diagnosis of parasitosis in developing countries is severely hampered due to the lack of trained parasitosis microscopy specialists (Saeed and Jabbar, 2018). For molecular methods, the PCR-based tools offer diagnosticians with high sensitivity and accuracy (Djohan et al., 2015; Lempereur et al., 2017; Llewellyn et al., 2016). However, this method is not without its challenges. It is labor-intensive, time-consuming, and prone to errors such as misdiagnosis and missed cases, largely due to the reliance on skilled technicians (Ricciardi and Ndao, 2015; Weerakoon and McManus, 2016). The limitations of traditional diagnostic methods highlight the urgent need for innovative solutions that can enhance diagnostic accuracy and efficiency.

Artificial intelligence (AI) has emerged as a promising technology in various medical fields, demonstrating significant potential in diagnostic applications. By utilizing advanced algorithms, including deep learning techniques, AI can process and analyze complex datasets with remarkable speed and accuracy (He et al., 2019). AI-assisted diagnostic tools have been successfully applied to CT image recognition of COVID-19 (Li et al., 2020), the automatic analysis of microscope slide images (Smith et al., 2018) and association of genome sequencing and proteomic profiles with pathogen phenotypes (Jamal et al., 2020; Lupolova et al., 2019). These advancements suggest that AI could revolutionize parasitic disease diagnosis, making it more accessible, especially in resource-limited settings.

This study makes several significant contributions: Adaptation of YOLOv4 for Parasitology: By applying the YOLOv4 object detection algorithm to recognize and classify parasitic helminth eggs, this work advances AI’s application in parasitology. This adaptation demonstrates that a widely used object detection model can be tailored to the unique morphology of parasite eggs, which are often challenging to distinguish manually. Evaluation Across Single and Mixed Species Samples: Unlike previous studies that primarily focused on single-species samples, this work explores both single and mixed species, revealing insights into the model’s robustness and limitations in handling complex, real-world diagnostic scenarios. Comprehensive Accuracy Analysis: By achieving high detection accuracy rates (e.g., 100% for *Clonorchis sinensis* and *Schistosoma japonicum*), this study provides an accuracy benchmark for future AI-based parasitology diagnostics, establishing a baseline for ongoing model improvements. Potential for Resource-Limited Settings: This research underscores AI’s capacity to reduce reliance on expert microscopy, making parasitic disease diagnosis more accessible. Such advancements could transform diagnostics in areas with limited access to specialized technicians, aligning with global health efforts to combat parasitic infections.

In this study, we aim to leverage the You Only Look Once (YOLOv4) object detection algorithm to detect and classify the morphology of human parasitic eggs, specifically focusing on nine common helminths: *A*. *lumbricoides*, *T*. *trichiura*, *E*. *vermicularis*, *A*. *duodenale*, *S*. *japonicum*, *P*. *westermani*, *F*. *buski*, *C*. *sinensis*, and *T*. spp. We evaluated and analyzed the recognition accuracy of our detection platform for both single and mixed egg specimens. Our findings indicate that this AI-assisted platform exhibits high efficiency and accuracy in identifying and classifying human parasites.

By integrating AI technology into parasitic disease diagnostics, this research aims to reduce the dependency on professional expertise, thereby enhancing diagnostic capabilities in both clinical and field settings. Ultimately, the rapid, accurate identification of parasitic infections will contribute significantly to the global efforts against parasitosis, providing a vital tool for public health.

## 2 Materials and methods

### 2.1 Sample collection and preparation

**Figure 1:**
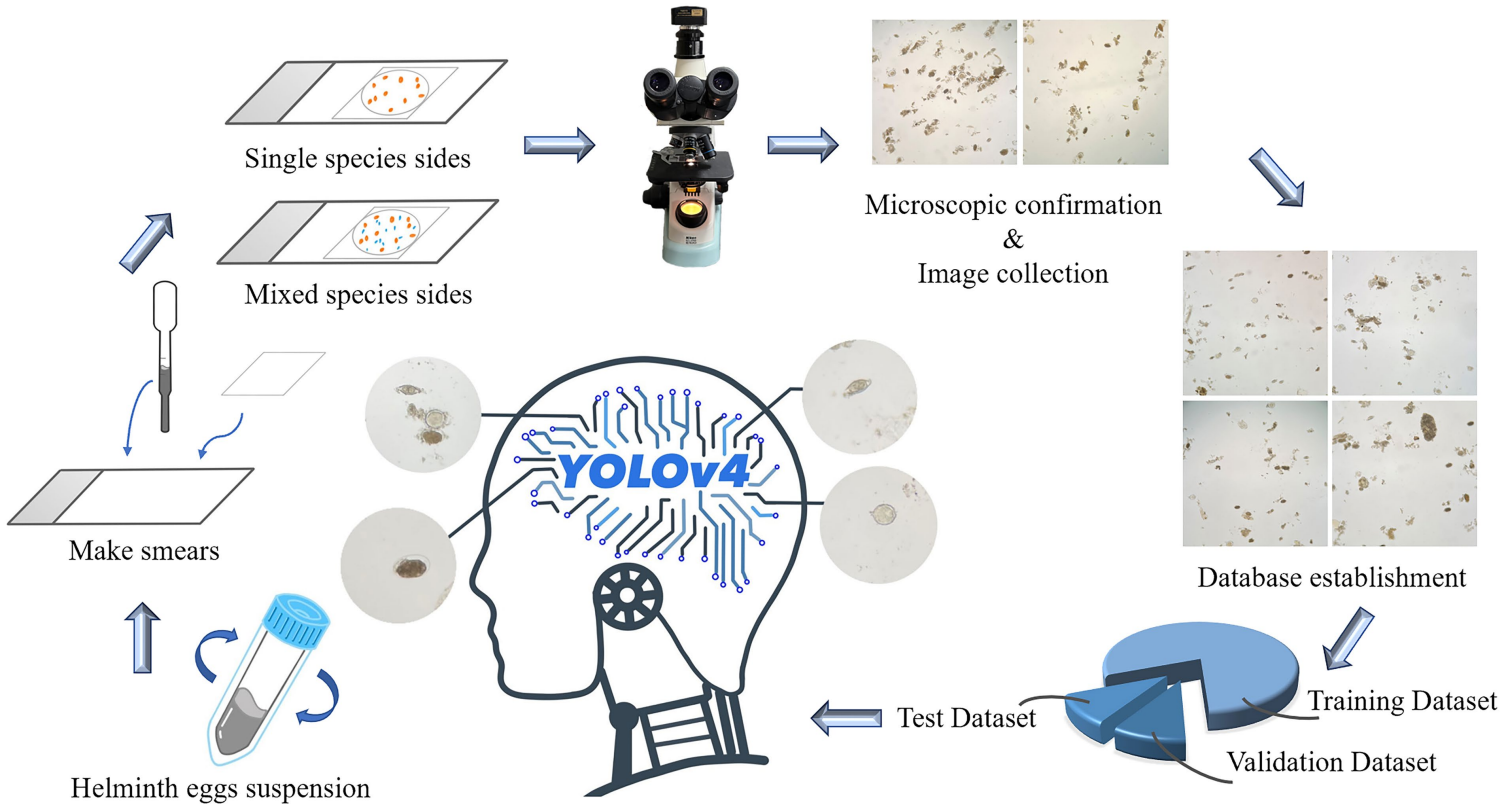
The collected egg suspensions were made into single species egg smears and mixed species smears, and the obtained images were classified and establish a dataset. The ratio of training set, verification set and test set was 8:1:1, and then YOLOv4 recognition model was established.

All helminth egg suspensions, including *A*. *lumbricoides*, *T*. *trichiura*, *E*. *vermicularis*, *A*. *duodenale*, *S*. *japonicum*, *P*. *westermani*, *F*. *buski*, *C*. *sinensis*, and *Taenia* spp., were purchased from Deren Scientific Equipment Co. Ltd. (Charoensuk et al., 2019). Two drops of vortex-mixed egg suspension (approximately 10 μL) were taken on a slide and covered with a coverslip (18 mm × 18 mm), taking care to avoid air bubbles, and the species of eggs were confirmed under the microscope. Subsequently, the eggs of *A*. *lumbricoides* and *T*. *trichiura*, eggs of *A*. *lumbricoides*, *T*. *trichiura*, and *A*. *duodenale*, eggs of *C*. *sinensis* and *Taenia* spp. were mixed and named Group 1, 2, and 3, respectively. All these sample slides were photographed via a light microscope (Nikon E100). As shown in the Figure 1.

### 2.2 Data collection and preprocessing

For YOLOv4, the dataset is divided into a training set, a validation set and a test set at a ratio of 8:1:1. The training set data are used to train the classification model, the validation set data are used to adjust the parameters of the model and optimize the model, and the test set data are used to test the classification performance of the model. Since the background color of the original image is inconsistent, we need to consider images with different background colors when selecting the test set to improve the reliability of the classification results.

For image cropping, an appropriate window size is selected by using a sliding window-like method, and the program is written to automatically crop one original image into 20 small images of the same size with a step size of the window size, with the size of each image being 518 × 486, which facilitates detection in the model.

## 3 Model selection

### 3.1 Parameter settings and evaluation metrics

#### 3.1.1 Training parameter settings

For YOLOv4, it was conducted using the Python 3.8 programming environment and the PyTorch framework running on an NVIDIA GeForce RTX 3090 GPU. During training, the images are compressed to a specific size. The k-means algorithm is initially employed for clustering to determine new anchor sizes. Mosaic data augmentation and mixup data augmentation are used for sample expansion. The initial learning rate is set to 0.01 with a learning rate decay factor of 0.0005. The Adam optimizer is utilized with a momentum value of 0.937, and the BatchSize is set to 64. A total of 300 epochs are trained, with the backbone feature extraction network frozen for the first 50 epochs to expedite convergence. If there is no further improvement in model performance after 200 epochs, the training is automatically stopped to save time (Supplementary Figure 1). The model is trained using the training set, and parameter optimization is performed using the validation set. Finally, the best model weights are output and saved. These weights are then used to predict the location and classify parasites in images.

#### 3.1.2 Evaluation metrics

Similar to other machine learning models, in object detection, there are true positives (TP), true negatives (TN), false positives (FP), and false negatives (FN) for the predicted results. To calculate the mean average precision (MAP), first, the recall (R) and precision (P) are computed. Recall reflects the cases of missed parasite detection, and its calculation formula is as follows.


Recall=TPTP+FN

Precision reflects cases of false parasite detection, and its calculation formula is as follows.


Precision=TPTP+FP

Object detection algorithms typically use average precision (AP) and mean average precision (MAP) to assess the accuracy of model detection results. The AP measures the detection accuracy for a single target class by evaluating the trade-off between precision and recall. The MAP represents the mean of all class AP values and is used to evaluate multiclass detection accuracy. It offers insight into the model’s performance across all classes. The calculation formula for MAP is as follows.


$$AP=\overset{1}{\underset{0}{\int }}\mathrm{PRdR}$$

$$\mathrm{MAP}=\frac{1}{C}\underset{C_{i}\in C}{\sum }AP_{\left(C_{i}\right)}$$

## 4 Results

### 4.1 General information

A total of 2083 images containing helminth eggs were obtained from the scanned slides. Of these images, 467 were mixed helminth egg images, which were used as the testing dataset.

### 4.2 Recognition of single species parasitic helminth eggs

**Figure 2:**
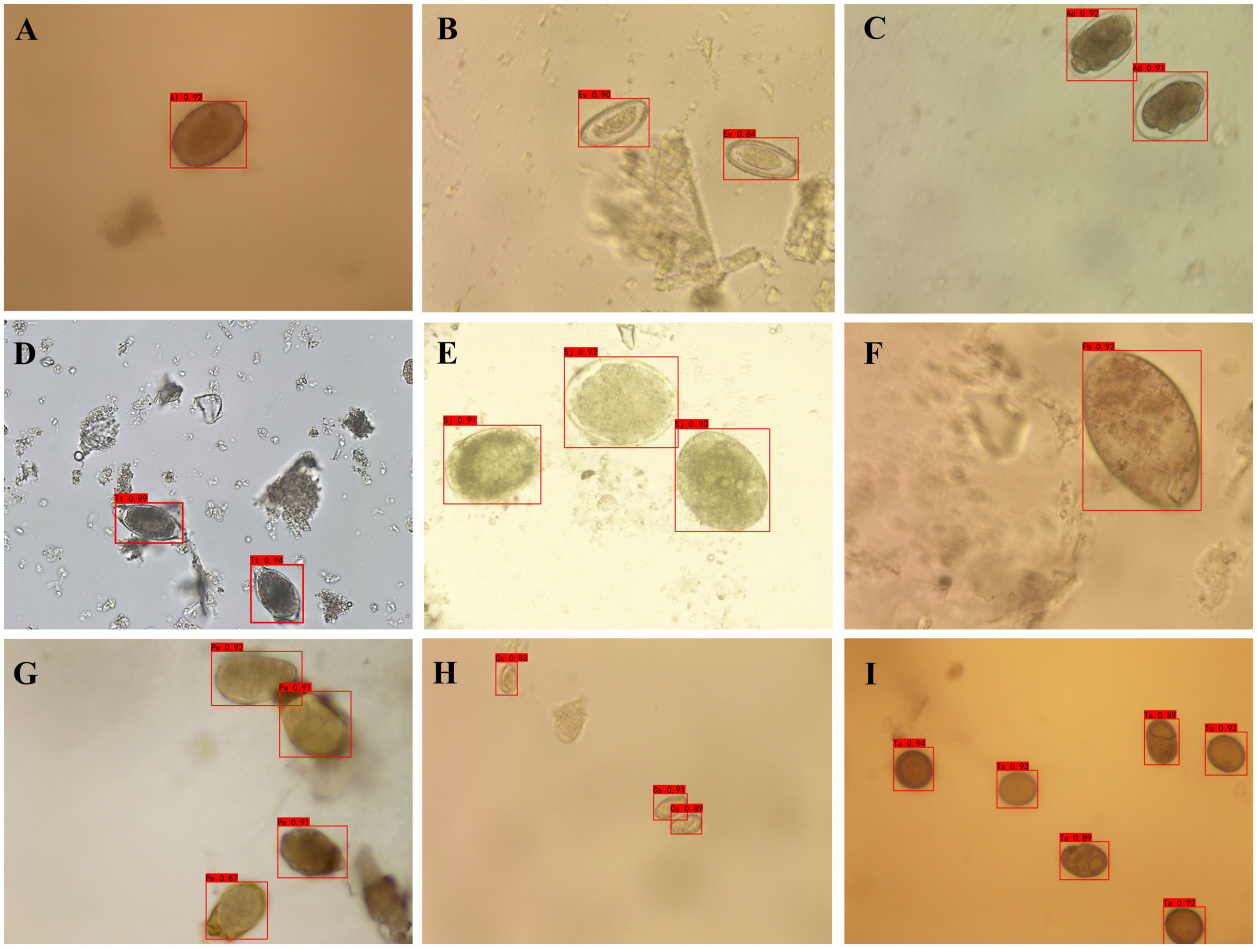
Detection outcomes of human helminth eggs using YOLOv4. **(A)** Fertilized eggs of *Ascaris Lumbricoides*; **(B)** Eggs of *Enterobius vermicularis*; **(C)** Eggs of *Ancylostoma duodenale*; **(D)** Eggs of *Trichuris trichiura*; **(E)** Eggs of *Schistosoma japonicum*; **(F)** Eggs of *Fasciolopsis buski*; **(G)** Eggs of *Paragonimus westermani*; **(H)** Eggs of *Clonorchis sinensis*; **(I)** Eggs of *Taenia* spp.

The optimized YOLOv4 model was employed to detect individual human helminth eggs. For individual eggs of a single species, the accuracy rates were 100.00% for *C*. *sinensis*, 100.00% for *S*. *japonicum*, 95.24% for *A*. *lumbricoides*, 95.24% for *A*. *duodenale*, 92.59% for *Taenia* spp., 89.31% for *E*. *vermicularis*, 88.00% for *F*. *buski*, 85.19% for *P*. *westermani*, and 84.85% for *T*. *trichiura* as depicted in Figure 2 and Table 1.

**Table 1:** Recognition results of human helminth eggs by YOLOv4.

Class	Parasitic helminth eggs	MAP	Recall	Precision
*Nematode*	*Ascaris lumbricoides*	100.00%	100.00%	95.24%
	*Enterobius vermicularis*	93.88%	95.83%	89.31%
	*Ancylostoma duodenale*	100.00%	100.00%	95.24%
	*Trichuris trichiura*	95.99%	93.33%	84.85%
*Trematoda*	*Schistosoma japonicum*	100.00%	100.00%	100.00%
	*Fasciolopsis buski*	99.45%	100.00%	88.00%
	*Paragonimus westermani*	93.41%	85.19%	85.19%
	*Clonorchis sinensis*	100.00%	100.00%	100.00%
*Cestoidea*	*Taenia* spp.	99.73%	96.15%	92.59%

Mean average precision (MAP); Taenia solium and Taenia seginata eggs are difficult to distinguish under the microscope, and they are collectively referred to as Taenia spp. in this study.

### 4.3 Recognition of mixed species parasitic helminth eggs

**Figure 3:**
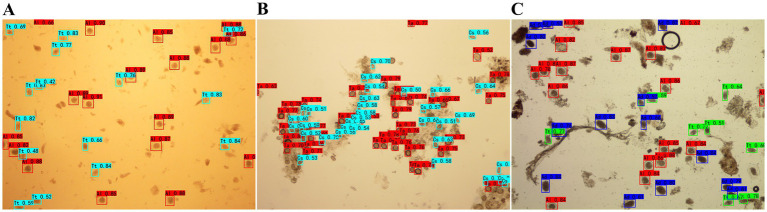
Detection outcomes of mixed human helminth eggs using YOLOv4 **(A)** Eggs of *A. lumbricoides* and *T*. *trichiura*; **(B)** Eggs of *Taenia* spp. and *Clonorchis sinensis* **(C)** Eggs of *A*. *lumbricoides*, *T*. *trichiura*, and *A*. *duodenale*.

In order to further verify the model, the mixture of eggs from different species were used for testing. For mixed helminth eggs, the accuracy rates for Group 1 (*A*. *lumbricoides* and *T*. *trichiura*) were 98.10 and 95.61%; For Group 2 (*A*. *lumbricoides*, *T*. *trichiura*, and *A*. *duodenale*), they were 94.86, 93.28, and 91.43%, and for Group 3 (*C*. *sinensis* and *Taenia* spp.), they stood at 93.34 and 75.00%, as illustrated in Figure 3 and Table 2.

**Table 2:** Recognition of mixed helminth eggs by YOLOv4.

Group	Parasitic helminth eggs	MAP	Recall	Precision
Group 1	*Trichuris trichiura*	99.01%	97.48%	98.10%
	*Ascaris lumbricoides*	96.84%	93.16%	95.61%
Group 2	*Trichuris trichiura*	97.28%	94.86%	94.86%
	*Ascaris lumbricoides*	90.73%	48.03%	93.28%
	*Ancylostoma duodenale*	96.39%	90.14%	91.43%
Group 3	*Clonorchis sinensis*	86.61%	82.01%	93.34%
	*Taenia* spp.	66.58%	55.81%	75.00%

Mean average precision (MAP).

## 5 Discussion

Parasitosis, caused by the invasion of parasites into the human body, leads to various pathological changes depending on the species and parasitic sites, making accurate diagnosis critical. The current gold standard for parasitic disease diagnosis is manual microscopy; however, this method is time-consuming, prone to error, and heavily reliant on experienced technicians. It is also lack of objectivity and accuracy and poor repeatability (Shen et al., 2016). This study aimed to enhance diagnostic efficiency by employing YOLOv4, a deep learning algorithm, to automatically recognize and classify parasite eggs in human fecal microscopic images. While the YOLOv4-based model demonstrated promising results, with high accuracy for some parasites, variability in detection accuracy was observed across species. For example, the model achieved 100% accuracy for *Clonorchis sinensis* and Schistosoma japonicum, but lower rates for species like *E*. *vermicularis* (89.31%), *F*. *buski* (88.00%), *P*. *westermani* (85.19%), and *T*. *trichiura* (84.85%).

In comparison, our model’s accuracy for detecting *Clonorchis sinensis* and *Schistosoma japonicum* was 100%, which outperforms the results from Lee et al. (2022) in their Helminth Egg Analysis Platform (HEAP), which reached 91.2% for *C*. *sinensis* using Faster R-CNN. Similarly, our model demonstrated higher accuracy than the YOLOv5-based approach in Huo et al. (2021), which achieved an average accuracy of 89.4% for single-species detection but struggled in distinguishing overlapping or mixed infections. The YOLOv4 model in this study provides an advantage in terms of sensitivity and specificity for these specific parasite types, likely due to its high detection resolution and optimized training process.

However, for mixed infections, our model’s accuracy varied. For instance, Group 1 (*A*. *lumbricoides* and *T*. *trichiura*) achieved high recognition rates of 98.10 and 95.61%, respectively, showing an improvement over HEAP’s mixed infection detection rate of 85.5%. Yet, accuracy dropped to 75% for Group 3 (mixed *C*. *sinensis* and *Taenia* spp.), revealing the model’s limitations in complex, overlapping scenarios. This drop aligns with the challenges noted in previous studies, such as the 79% accuracy reported in Huo et al. (2021) for similar overlapping cases, which emphasizes that mixed infections remain a difficult task for automated detection systems. Given that traditional microscopy can provide accurate diagnoses when performed by skilled technicians, it remains a robust diagnostic tool. However, AI-based methods offer distinct advantages in terms of speed and automation, especially in resource-limited settings where trained professionals may not always be available.

Several factors can significantly influence the performance of AI-assisted parasite detection. The quality of sample preparation is crucial; improperly prepared samples can obscure egg morphology, leading to misclassification. Additionally, the size of parasitic eggs can affect recognition accuracy, as variations can complicate the detection algorithms. Furthermore, preservation methods play a vital role; poor preservation can cause morphological changes that hinder accurate recognition. Addressing these factors is essential for enhancing the reliability of AI diagnostics.

A key point for determining whether AI-based image analysis is superior to traditional methods lies in statistical validation. Statistical analysis comparing the AI model’s performance to microscopy and other diagnostic tools (such as PCR or immunological assays) would be essential for drawing firm conclusions. This analysis would quantify whether the AI system offers statistically significant improvements in accuracy, sensitivity, and specificity. For instance, a comprehensive study comparing the false-positive and false-negative rates between AI-based diagnostics and traditional methods would highlight the strengths and weaknesses of each approach. While the overall recognition accuracy of 94.41% in our model is promising, it still falls short of the ideal accuracy needed for a diagnostic tool to fully replace microscopy in complex cases, particularly in mixed infections.

As seen in previous studies, such as the Helminth Egg Analysis Platform (HEAP) by Lee et al. (2022), challenges with overlapping eggs and underrepresented species also affect the performance of AI models. Huo et al. (2021) reported similar findings with YOLOv5, noting that while AI-based models exhibit higher speed and efficiency, they require further optimization to handle real-world variability in fecal samples. Both our study and theirs suggest that expanding training datasets, enhancing image augmentation techniques, and potentially integrating multi-model approaches (e.g., ensemble learning) could improve overall accuracy.

In conclusion, while AI-based models like YOLOv4 show significant potential to reduce the reliance on manual microscopy, especially in less specialized settings, they are not yet ready to completely replace traditional methods. Instead, hybrid approaches that combine the strengths of AI with expert review could be more effective in ensuring accurate diagnosis, particularly for parasites with lower detection accuracy or in complex infection scenarios. Addressing the factors influencing AI performance and further refinement of these technologies are critical steps toward improving the efficiency and reliability of parasitic disease diagnostics in the future.

## 6 Conclusion

The YOLOv4-based automatic recognition system for parasite eggs achieved an overall recognition accuracy of 94.41%, demonstrating significant potential to enhance the speed and efficiency of parasitic disease diagnosis. However, the variability in detection accuracy across different parasite species, particularly in mixed infections, highlights the model’s limitations. For example, accuracy rates for species like *E*. *vermicularis* (89.31%), *F*. *buski* (88.00%), *P*. *westermani* (85.19%), and *T*. *trichiura* (84.85%) were lower than expected, and in mixed infections such as Group 3 (mixed *C*. *sinensis* and *T*. spp.), the recognition accuracy dropped to 75%. These results indicate that while the AI model performs well for certain parasites, it struggles with complex diagnostic scenarios, such as mixed infections, where traditional microscopy still holds an advantage due to its ability to detect subtle variations in parasite morphology.

## Data Availability

The raw data supporting the conclusions of this article will be made available by the authors, without undue reservation.

## Supplementary material

SUPPLEMENTARY FIGURE 1 YOLO v4 structure (A) Overall structure. YOLOv4 is comprised of three main components: Backbone, Neck, and Head. (B) CSPNet structure. YOLOv4 employs CSPDarknet-53 as the primary feature extraction network, which involves modifications to the residual blocks of Darknet-53 and the integration of the CSPNet (Cross Stage Partial Networks) architecture. (C) ASPP Structure. ASPP (Atrous Spatial Pyramid Pooling) uses dilated convolutions with different dilation rates to capture multi-scale features while maintaining the spatial dimensions, thereby enhancing the network's ability to recognize objects of varying sizes. (D) SE Structure. The final feature layer incorporates Spatial Pyramid Pooling Layers (SPP Layers) and integrates the Squeeze-and-Excitation (SE) attention mechanism. This mechanism models inter-channel relationships through Squeeze and Extraction operations, enabling the model to adaptively learn the importance of each channel and assign varying weights based on task requirements.
