# Supplementary figures and images for: Recognition of parasitic helminth eggs via a deep learning-based platform
Source: Front Microbiol. 2024 Nov 20;15:1485001. doi: 10.3389/fmicb.2024.1485001 (PMC11614813; doi:10.3389/fmicb.2024.1485001)

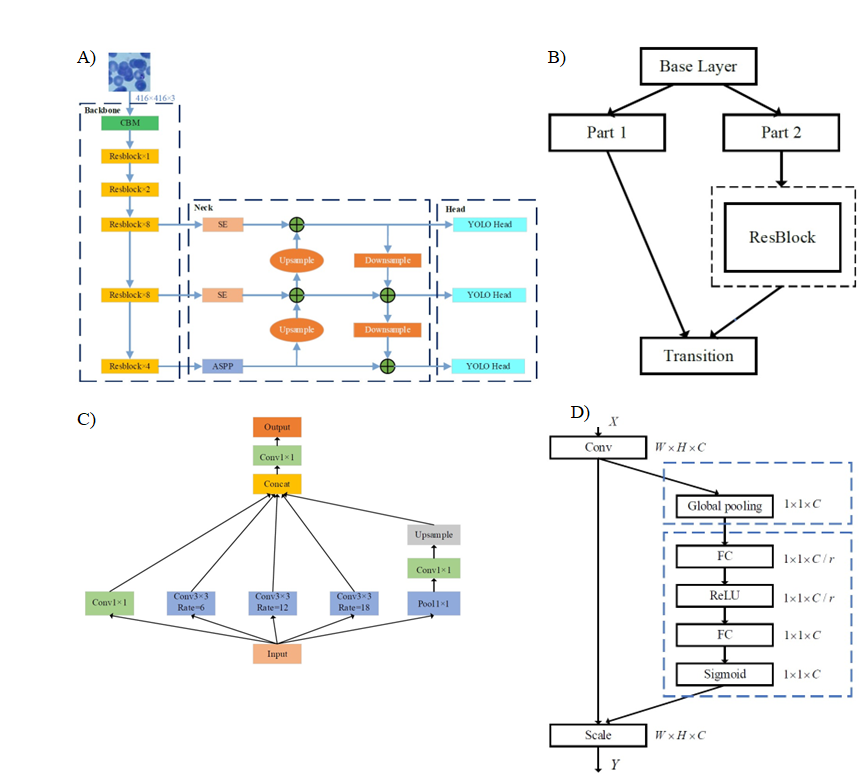

Supplement: SUPPLEMENTARY FIGURE 1 — YOLO v4 structure (A) Overall structure. YOLOv4 is comprised of three main components: Backbone, Neck, and Head. (B) CSPNet structure. YOLOv4 employs CSPDarknet-53 as the primary feature extraction network, which involves modifications to the residual blocks of Darknet-53 and the integration of the CSPNet (Cross Stage Partial Networks) architecture. (C) ASPP Structure. ASPP (Atrous Spatial Pyramid Pooling) uses dilated convolutions with different dilation rates to capture multi-scale features while maintaining the spatial dimensions, thereby enhancing the network's ability to recognize objects of varying sizes. (D) SE Structure. The final feature layer incorporates Spatial Pyramid Pooling Layers (SPP Layers) and integrates the Squeeze-and-Excitation (SE) attention mechanism. This mechanism models inter-channel relationships through Squeeze and Extraction operations, enabling the model to adaptively learn the importance of each channel and assign varying weights based on task requirements. [file Image_1.png]
